# Role of canonical and noncanonical autophagy pathways in shaping the life journey of B cells

**DOI:** 10.3389/fimmu.2024.1426204

**Published:** 2024-07-30

**Authors:** Yiwen Wang, Lan Wu, Luc Van Kaer

**Affiliations:** Department of Pathology, Microbiology and Immunology, Vanderbilt University School of Medicine, Nashville, TN, United States

**Keywords:** antibodies, autophagy, B cell biology, B lymphocytes, canonical autophagy, humoral immunity, noncanonical autophagy

## Abstract

Autophagy is a regulated intracellular catabolic process by which invading pathogens, damaged organelles, aggregated proteins, and other macromolecules are degraded in lysosomes. It has been widely appreciated that autophagic activity plays an important role in regulating the development, fate determination, and function of cells in the immune system, including B lymphocytes. Autophagy encompasses several distinct pathways that have been linked to B cell homeostasis and function. While B cell presentation of major histocompatibility complex (MHC) class II-restricted cytosolic antigens to T cells involves both macroautophagy and chaperone-mediated autophagy (CMA), plasma cells and memory B cells mainly rely on macroautophagy for their survival. Emerging evidence indicates that core autophagy factors also participate in processes related to yet clearly distinct from classical autophagy. These autophagy-related pathways, referred to as noncanonical autophagy or conjugation of ATG8 to single membranes (CASM), contribute to B cell homeostasis and functions, including MHC class II-restricted antigen presentation to T cells, germinal center formation, plasma cell differentiation, and recall responses. Dysregulation of B cell autophagy has been identified in several autoimmune and autoinflammatory diseases such as systemic lupus erythematosus, rheumatoid arthritis, and inflammatory bowel disease. In this review, we discuss recent advances in understanding the role of canonical and noncanonical autophagy in B cells, including B cell development and maturation, antigen processing and presentation, pathogen-specific antibody responses, cytokine secretion, and autoimmunity. Unraveling the molecular mechanisms of canonical and noncanonical autophagy in B cells will improve our understanding of B cell biology, with implications for the development of autophagy-based immunotherapies.

## 1 Introduction

B lymphocytes are the source of pathogen-specific antibodies, and thus, are critical players of the vertebrate immune system. Their capacity to generate long-lasting humoral immunity against microbial pathogens enables B cells to protect the host against repeated infections, which provides the mechanistic basis for vaccine efficacy.

B cells can be partitioned into two major subpopulations, namely B1 and B2 cells, each containing several subsets (1–5). B1 cells develop from precursors during the prenatal period and are largely maintained by self-renewal after birth. They display limited immunoglobulin receptor diversity and are enriched in the peritoneal and pleural cavities. These cells are the major source of so-called natural IgM antibodies that are constitutively secreted without prior immune activation. B1 cells can be further divided into B1a and B1b cells based on the presence or absence of the inhibitory surface receptor CD5. B1a cells produce antibodies that react with molecular features common to many microbes and also contribute to the removal of senescent cells (4). B1b cells contribute to the generation of adaptive immune responses against bacterial pathogens without the assistance of T cells, thus contributing to T cell-independent (TI) antibody responses. Antibodies produced by B1 cells tend to be of low affinity and are largely limited to the IgM and IgG3 subclasses due to their scarcity of affinity maturation and class switching, respectively. The contribution of B1 cells to immune memory remains unclear (5).

The B2 B cell lineage includes marginal zone B (MZB) cells and follicular B (FOB) cells (1–3). MZB cells are derived from the bone marrow and share a common bone marrow precursor with FOB cells, from which they diverge at the transitional T2 stage (6). MZB cells are predominantly generated early in life. They are long-lived and are found within the marginal zone of the spleen where they can rapidly respond to incoming antigens from the circulation. Similar to B1 cells, MZB cells exhibit innate-like properties and functions. They display a restricted B cell receptor (BCR) repertoire, can rapidly generate TI antibody responses of the IgM and IgG isotypes against blood-borne invaders, and have limited capacity to generate memory responses (7).

FOB cells, hereafter usually referred to as B cells, constitute the majority of the B cell population. These cells develop in the bone marrow with successive steps of assembling and expressing functional antigen-receptor genes. With the surface expression of a unique BCR, immature B cells are tested for autoreactivity following exposure to self-antigens in their environment. They then migrate to peripheral lymphoid organs where they go through transitional stages (T1-T3) before becoming mature, antigen-inexperienced B cells (8, 9). Following interaction with cognate antigen, activated B cells may differentiate into short-lived, antibody-secreting cells (ASCs). However, robust antibody and B cell memory responses require the participation of a specialized subset of major histocompatibility complex (MHC) class II-restricted CD4^+^ T cells called T follicular helper (T_FH_) cells. This process involves B cell internalization of BCR-bound antigens, followed by processing in intracellular vesicles and presentation of antigen-derived peptides by MHC class II to CD4^+^ T cells. Naïve B cells activated by T_FH_ cells proliferate and differentiate within a specialized anatomic structure of lymphoid organs called the germinal center (GC). Within GCs, B cells undergo somatic hypermutation that drives the generation of high-affinity antibody responses. B cells may also undergo class switch recombination (CSR), driven by CD4^+^ T cell produced cytokines, and this process may occur within or outside GCs. B cells eventually differentiate into antibody-secreting plasma cells or long-lived memory B cells (3, 10).

The life cycle of a B cell spans its genesis and maturation in the bone marrow, its survival as a mature naïve B cell in the periphery, its activation and proliferation in response to cognate antigen, its differentiation into an antibody-secreting plasma cell, and its acquisition of a memory phenotype. Each stage along the dynamic life cycle of a B cell is enabled by a bioenergetically unique profile of metabolites that facilitates its transition between non-cycling quiescent and rapidly proliferating states (1–3, 11). In dealing with these metabolic alterations and their associated stress, B cells have taken advantage of the fundamental cellular process called autophagy. Under the suppressive control of nutrient sensor mechanistic target of rapamycin (mTOR) and the activating control of the energy sensor AMP-activated protein kinase (AMPK), autophagy ensures the cellular availability of biomolecules required for B cell survival and function (12). Additionally, autophagy selectively degrades deleterious cytosolic entities and organelles (e.g., damaged mitochondria) during B cell activation (13–15). Interestingly, emerging evidence indicates that many autophagy factors also participate in processes that are related to yet clearly distinct from the classical autophagy pathway, including noncanonical autophagy (16). These noncanonical autophagy pathways are emerging as important contributors to B cell biology.

Here, we discuss how autophagy shapes B cell development, differentiation, fate, and function. We highlight exciting recent findings showing a critical role of noncanonical autophagy in B cell biology. We further aim to provide a perspective to reconcile and better understand discrepancies observed in early studies investigating the role of autophagy factors in B cell development and function.

While this review article focuses on B cells, autophagy plays a critical role in the development and function of many other immune cell types. We refer the readers to excellent prior articles focused on autophagy in T cells (12, 17, 18), macrophages (19, 20), and dendritic cells (21).

## 2 Autophagy

**Figure 1:**
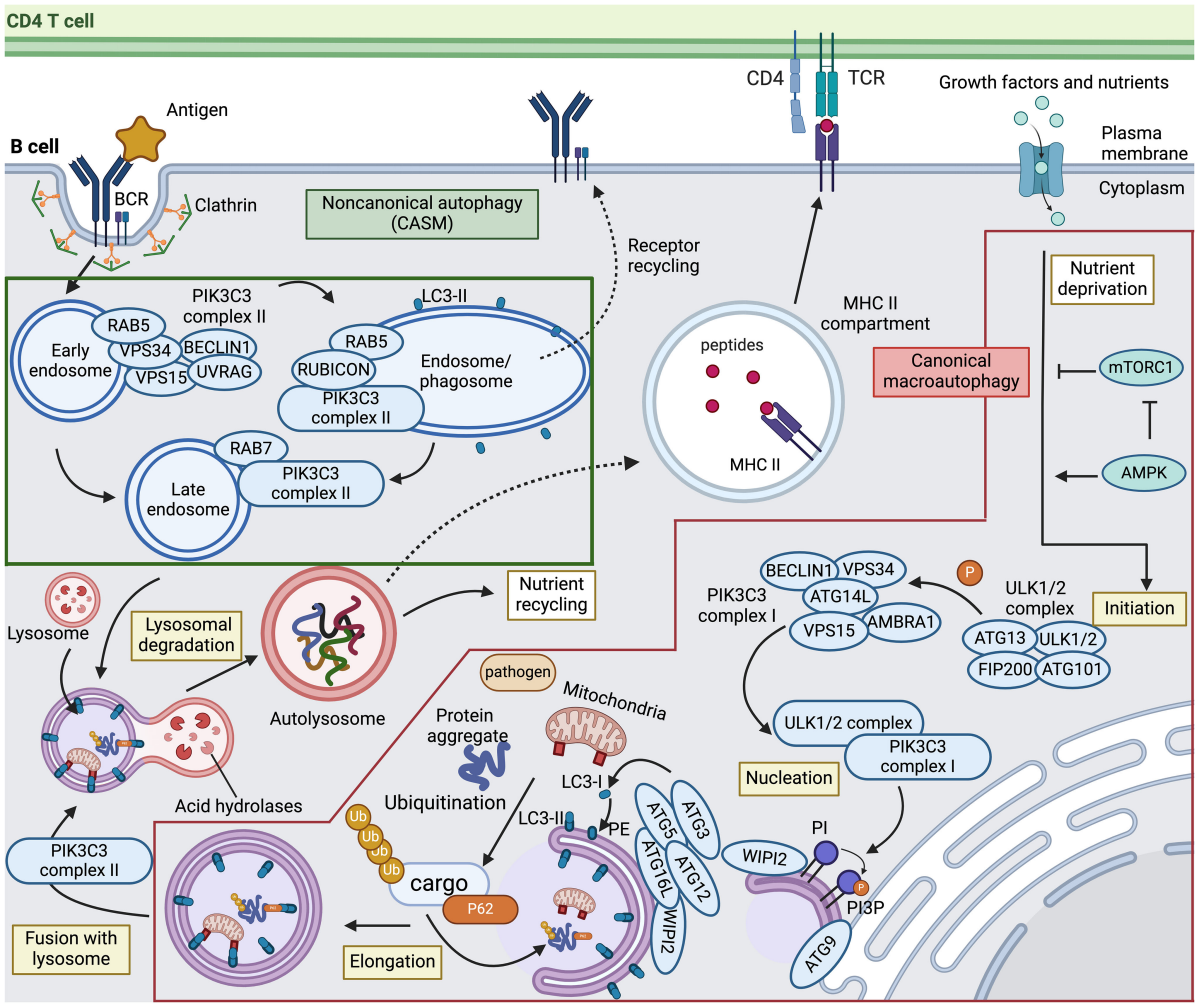
Canonical and noncanonical autophagy pathways in B cells. Canonical macroautophagy (indicated in the red box) is initiated when signals mediated by nutrient starvation or other stressors activate AMP-activated protein kinase (AMPK) and concurrently inhibit mechanistic target of rapamycin complex 1 (mTORC1), thereby prompting the formation of the ULK1/2 autophagy initiation complex. This complex then phosphorylates PIK3C3 complex I to promote phagophore nucleation. The ULK1/2 complex also recruits and activates transmembrane protein ATG9 to distribute lipids for autophagosome biogenesis from various membrane sources. Upon nucleation, the ATG12–ATG5–ATG16L1 conjugation system, recruited by WIPI2, functions as an E3-like ligase to mediate LC3 lipidation. The precursor of LC3 is cleaved into its mature form (LC3-I), followed by conjugation to phosphatidylethanolamine (PE) into LC3-PE (LC3-II). LC3 is critically involved in sequestering specifically labeled cargo into autophagosomes via cargo receptors. After maturation, autophagosomes fuse with the lysosome to form the autolysosome in a process involving PIK3C3 complex II. The sequestered cytoplasmic materials are then degraded via lysosomal hydrolases and recycled. The dashed line towards the MHC II compartment indicates the potential contribution of both macroautophagy and chaperone-mediated autophagy to the capacity of B cells to present antigens to CD4 T cells. Noncanonical autophagy, also called conjugation of ATG8 to single membranes (CASM) (indicated in the green box), is thought to play a critical role in antigen-activated B cells. Upon antigen engagement, the B cell receptor (BCR) undergoes clathrin-mediated internalization. PIK3C3 complex II that includes RUBICON is recruited to drive LC3 lipidation on the sealed phagosome and maturation of RAB5-positive early endosomes. Although the mechanisms of LC3-associated phagocytosis (LAP) and LC3-associated endocytosis (LANDO), indicated by dashed lines, remain unclear, these pathways may be involved in cargo degradation and recycling of receptors to the plasma membrane. TCR, T cell receptor; PI, phosphatidylinositol; PI3P, PI 3-phosphate. This figure was created by BioRender.com.

Autophagy is a conserved intracellular recycling process that delivers cytoplasmic material to lysosomes for degradation (22, 23). Classical or canonical autophagy processes are classified into three pathways depending on the type of cargo delivered to the lysosome: macroautophagy, microautophagy, and chaperone-mediated autophagy (CMA) (24, 25). Macroautophagy (hereafter usually referred to as autophagy) is the best studied form that can be subdivided into five phases (**Figure 1**): a) initiation by nutrient stress, b) nucleation of the isolation membrane, c) elongation and sealing of the double membrane to sequester cargo, d) fusion of the autophagosome and lysosome, and e) lysosomal degradation of the vesicle substrates (26).

Bioenergetic stress imposed by starvation results in AMPK activation and mTORC1 inactivation, which directly or indirectly initiates autophagic cascades involving multiple protein complexes (27, 28). Starting with autophagy initiation, the UNC51-like autophagy-activating kinase (ULK)1/2 complex is a multiprotein complex consisting of ULK1, ULK2, FAK family-interactive-protein of 200 kDa (FIP200/RB1CC1), autophagy-related (ATG) protein 13 (ATG13) and ATG101. The ULK1/2 complex directly activates the class III phosphatidylinositol (PtdIns) 3-kinase (PI3K) catalytic subunit 3 (PIK3C3) complex I, which produces a pool of PtdIns 3-phosphate (PtdIns3P) that triggers nucleation of the phagophore. This PIK3C3 complex I consists of PIK3C3/vacuolar protein sorting 34 (VPS34), BECLIN1, protein kinase-like VPS15, ATG14L, nuclear receptor-binding factor 2 (NRBF2), and activating molecule in BECLIN1-regulated autophagy (AMBRA) (29–31). ULK1/2 also recruits and phosphorylates transmembrane proteins, including ATG9, to distribute lipids for autophagosome biogenesis from membrane sources such as the endoplasmic reticulum (ER), mitochondria, and plasma membrane (32). Following nucleation, two essential ubiquitin-like conjugation systems are activated for membrane expansion and fusion. First, WD repeat domain phosphoinositide-interacting 2 (WIPI2) recruits ATG16L1 and forms the ATG12-ATG5:ATG16L1 complex, sequentially catalyzed by the E1-like ubiquitin enzyme ATG7 and the E2-like ubiquitin enzyme ATG10. This complex then acts as an E3-like ubiquitin ligase that leads to the lipidation of ATG8/microtubule-associated protein light chain 3 (LC3) family proteins. ATG4, ATG7, and ATG3 cooperate to cleave the precursors of ATG8/LC3-like proteins into their mature forms (termed LC3-I), followed by conjugation to phosphatidylethanolamine (PE) into LC3-PE (termed LC3-II) and recruitment to autophagosomes with the support of WIPI2 (33–36). The conversion of LC3-I to LC3-II, measured in the presence of lysosomal protease inhibitors, is often employed as an approach to monitor autophagy (37, 38). While this process may non-selectively degrade all targets in response to stressors such as starvation, selective forms of autophagy may specifically target ubiquitinated cargo with the help of ATG8/LC3 and autophagy receptors such as P62/SQSTM1 (39). Upon completion of phagophore expansion and closure, the resulting double-membraned autophagosome vesicle fuses with a lysosome to generate an autolysosome, facilitated by regulators controlling endosome maturation and vesicle trafficking (29). Such regulators include cytoskeleton components and related motor proteins, endosomal sorting complexes required for transport (ESCRT) components, small guanosine triphosphatases (GTPases), including RAS-related protein RAB7, and soluble *N*-ethylmaleimide-sensitive factor attachment protein receptor (SNARE) complexes (40–42). Once the enclosed contents are exposed to lysosomal products, lysosomal hydrolases mediate substrate degradation and metabolite recycling.

Employing different pathways to route cytoplasmic materials for lysosomal degradation, microautophagy directly sequesters small cytosolic cargos with endosomes or lysosomes, whereas CME is mediated by cytosolic chaperones to deliver cytosolic cargos to lysosomes for selective degradation (43).

## 3 Noncanonical autophagy

Recent research has provided evidence that classical autophagy factors are also engaged in several related yet distinct pathways, collectively called noncanonical autophagy (23, 43). Distinct from the double-membrane structures formed in canonical autophagy, one feature of noncanonical autophagy is the formation of single-membrane vesicles within the endolysosomal system for ATG8/LC3 conjugation and lysosomal degradation (**Figure 1**). These noncanonical autophagy pathways are now collectively referred to as conjugation of ATG8 to single membranes (CASM) (44). These two terms, noncanonical autophagy and CASM, will be used interchangeably in this review. Associated with this distinctive feature, CASM is independent of some of the upstream autophagy machinery, including ULK1/2, FIP200, ATG13, ATG9, WIPI2, and ATG14L, but requires the core ubiquitin-like conjugation system that supports ATG8/LC3 lipidation to the membrane, including ATG3, ATG4, ATG5, ATG7, ATG10, ATG12, and ATG16L1 (31, 45). Another pivotal dissimilarity is that CASM requires PIK3C3 complex II, with a different composition compared to the canonical autophagy PIK3C3 complex I. While both complexes employ PIK3C3/VPS34 and VPS15, PIK3C3 complex II is composed of ultraviolet radiation resistance-associated gene protein (UVRAG) and Run domain BECLIN1-interacting and cysteine-rich containing protein (RUBICON), instead of BECLIN1, AMBRA and ATG14L (30). Of note, two isoforms of RUBICON with distinguished size, ~130 kDa and ~100 kDa, have been identified and show opposing roles for autophagy regulation (46). In addition, PIK3C3 complex II locates to the phagophore/endosome after cargo encapsulation and vesicle sealing whereas PIK3C3 complex I is recruited to the ER for autophagosome biogenesis during canonical autophagy (30).

Several recent findings have deepened the understanding of CASM, especially the fundamental process involving autophagosome fusion with vesicles within the endocytic network of phagocytosis or endocytosis. Both pathways involve conjugation of ATG8/LC3 to single-membranes, and these processes are thus termed LC3-associated phagocytosis (LAP) and LC3-associated endocytosis (LANDO) (45, 47, 48). The latter pathways, as well as other CASM pathways, are discussed in more detail below.

### 3.1 LC3-associated phagocytosis and endocytosis

In LAP, LC3 lipidation on the outer face of the phagosome membrane requires the production of nicotinamide adenine dinucleotide phosphate (NADPH) oxidase-2 (NOX2)-induced reactive oxygen species (ROS). Under these conditions, LC3 promotes phagosome closure and phagosome-lysosome fusion, leading to degradation of the engulfed material (49). Macrophages with LAP-deficiency display impaired phagocytosis, highlighting the crucial role of LAP in the effective clearance of phagocytic cargo (50). In contrast to LAP, the specific role of LC3 lipidation in the context of LANDO is poorly defined. LANDO is characterized by conjugation of LC3 to clathrin- and RAB5-positive endosomes (51–53). Typically, endocytic pathways are categorized as clathrin-dependent or -independent, both relying on PIK3C3 complex II (30). In contrast to LAP, the absence of LC3 conjugation in LANDO does not result in defects in cargo degradation but instead causes reduced cargo receptor surface recycling (54). Subsequent sections will explore the impact of LAP and LANDO on B cell biology.

### 3.2 Other CASM pathways

In addition to its established role in heterophagy, CASM controls extracellular secretion. In the LC3-dependent extracellular vesicle loading (LDEL) pathway, LC3 and LC3-conjugation machinery mediate the loading of cytosolic cargos, including RNA binding proteins and RNA, into extracellular vesicles (EVs) for secretion outside cells (55, 56). Genetic evidence also implicates ATG factors to participate in unconventional secretion of acyl-CoA-binding protein Acb1 in yeast, several inflammatory cytokines in macrophages, and plasma membrane trafficking of membrane proteins in HEK293 cells (57–60). Despite limited evidence correlating CASM-mediated exocytosis to B cells, it is tempting to speculate that this secretory pathway may play a role in B cell function. The mechanism by which secretory CASM may relate to B cell function will be further explored in subsequent sections.

## 4 Autophagy in B cell biology

**Figure 2:**
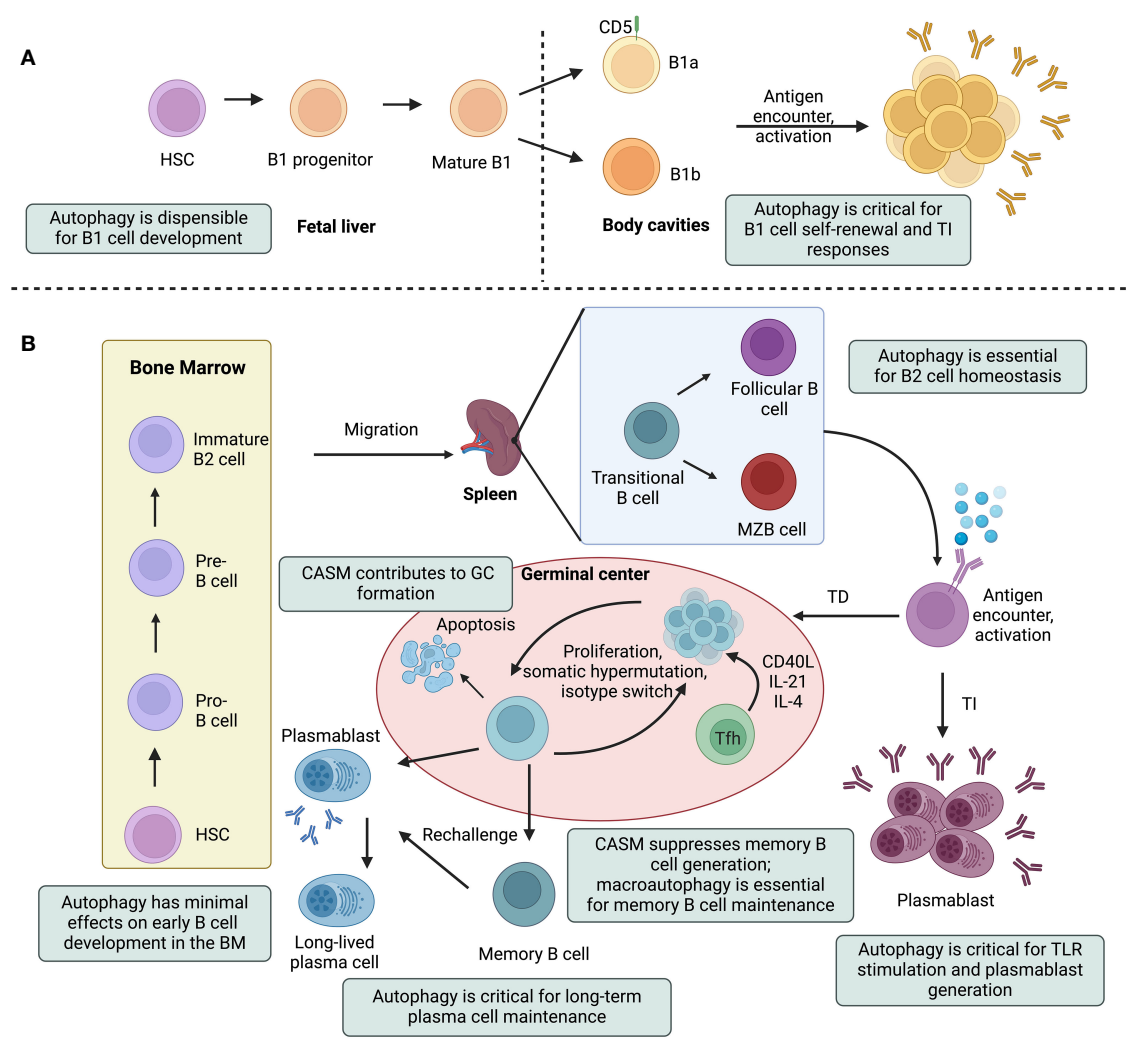
Canonical and noncanonical autophagy pathways impinge on multiple steps in the life of a B cell. A simplified outline of B cell lineage differentiation is depicted, with a focus on the effects of autophagy on B cell development, maturation, and effector functions. Hematopoietic stem cells (HSCs) give rise to the progenitors of two major subtypes of B lymphocytes: innate-like B1 cells **(A)** and B2 cells **(B)**. B1 cell generation is restricted to fetal life and these cells then undergo self-renewal in the periphery. B1 cells can be further subdivided into B1a and B1b cells, based on the expression of the CD5 surface marker. Autophagy-deficient B1a cells fail to self-renew due to dysregulation of lipid and mitochondrial homeostasis. B2 cells, on the other hand, develop in the bone marrow (BM) and mature in peripheral lymphoid tissues. B2 cell development in the BM progresses through the pro-B cell, pre-B cell and immature B cell stages. Immature B cells that successfully express nonself-reactive B cell receptors leave the BM, become transitional B cells, and mature into follicular B (FOB) or marginal zone B (MZB) cells in secondary lymphoid tissues (i.e., lymph nodes and spleen). Depending on the antigen they encounter, mature B cells may engage in either a T cell-dependent (TD) or T cell-independent (TI) antibody response. B cells, mostly MZB cells, undergo TI responses and rapidly differentiate into short-lived plasmablasts that secrete IgM antibodies. Under certain activation conditions, these short-lived plasmablasts require autophagy to alleviate adverse effects of ER stress and mitochondrial damage. During TD responses, activated B cells form structures called germinal centers (GCs) and undergo differentiation, somatic hypermutation, and class switch recombination. Eventually, these cells will differentiate into antibody-secreting plasma cells or long-lived memory B cells. Conjugation of ATG8 to single membranes (CASM) promotes the formation of high-affinity GC B cells. The unfolded protein response in plasma cells induces autophagy, which is crucial for the maintenance of plasma cells and antibody production. Memory B cell generation is inhibited by CASM, but relies on canonical autophagy that protects against mitochondrial damage. Tfh, T follicular helper; TLR, toll-like receptor. This figure was created by BioRender.com.

Autophagy in B cells is a dynamic process that varies based on the stage of B cell maturation and the particular B cell subset. **Figure 2** summarizes the stages in the life of B cells that are impacted by canonical or noncanonical autophagy. Many of these studies were performed by analyzing the B cell phenotypes of mice genetically deficient in specific ATG factors, as summarized in **Table 1**. These studies are discussed in more detail in the following sections.

**Table 1:** Effects of B cell autophagy factor-deficiency on B cell development, homeostasis, differentiation and function.

Autophagy factor(s)^1^	Mouse model^2^	B cell phenotype	Ref.
BECLIN1	Inject *Beclin1^-/-^* embryonic stem cell clones into *Rag* ^-/-^ blastocysts to obtain chimeras	• B cell development and maturation: ◦ Decreased prevalence of early B cell compartment in BM ◦ Diminished T2 B cell levels and normal MZB cell levels ◦ Normal development of peritoneal B1 cells • *In vitro* stimulation: ◦ Normal proliferation in response to LPS + IL-4	(61)
ATG5	Inject *Atg5^-/-^* fetal liver cells into irradiated *Rag^-/-^* mice	• B cell development and maturation: ◦ Decreased level of pre-B cells and mature circulating B cells (Hardy fractions^3^ D and F) in the BM, accompanied by increased cell death ◦ Decreased number of B1 and B2 cells and normal B2 subsets in spleen ◦ Decreased number of B1 and B2 cells in peritoneum	(62)
	*Atg5^f/f^;CD19-Cre*	• B cell development and maturation: ◦ Reduced number of mature circulating B cells (Hardy fraction F) ◦ Comparable number of B2 cells in spleen, and fewer B cells in GALT ◦ Decrease in peritoneal B1a cells • *In vitro* stimulation: ◦ Impaired IgM secretion and PC transcription factor upregulation after LPS or CpG stimulation without affecting viability • Antibody responses: ◦ Normal level of steady state IgM, IgG, and IgA in serum ◦ TD antigen: TNP-CGG + Alum ▪ Lower level of Ag-specific serum Ig ▪ Decreased number of ASCs ◦ TD antigen: *Heligmosomoides polygyrus* ▪ Decreased level of IgG and IgE in serum ◦ TI antigen: TNP-LPS/Ficoll ▪ Lower level of Ag-specific serum Ig • Intestinal inflammation model induced by DSS: ◦ Impaired secretion of IgA ◦ Less significant weight loss	(62, 63)
	*Atg5^f/f^;CD19-Cre*	• *In vitro* stimulation: ◦ Larger ER and enhanced ER stress in response to LPS stimulation ◦ Enhanced production of BLIMP1 and Ig with LPS stimulation, yet reduced cell viability • Antibody responses: ◦ TD antigen: NP-CGG + Alum ▪ Normal GC formation ▪ Lower level of serum Ig ▪ Lower frequency of long-lived PC at 11 months after immunization ◦ TI antigen: pneumovax ▪ Less production of Ig ◦ TI antigen: NP-Ficoll ▪ Higher level of Ag-specific lg in the serum	(64)
	*Atg5^f/f^;CD19-Cre*	• *In vitro* stimulation: ◦ Proliferation of MZB and peritoneal B1 cells increased in response to TLR stimulation, with increased activation of MAPK, IKK, and NF-κB	(65)
	*Atg5^f/f^;Mb1-Cre*	• B cell development and maturation: ◦ Increased frequency of pre-B/immature B cells and reduced frequency of mature circulating B cells in the BM ◦ Homeostatic defect of FOB survival in spleen ◦ Decreased proportion of B1a and B2 cells in the peritoneum • *In vitro* stimulation: ◦ Normal B cell proliferation and survival under anti-IgM stimulation with or without anti-CD40 ◦ Decreased viability after LPS stimulation accompanied by mitochondrial damage • Antibody responses: ◦ TD antigen: OVA + CFA ▪ Normal level of total Ig but reduced Ag-specific IgM in the serum	(66)
	*Atg5^f/f^;CD21-Cre*	• B cell development and maturation: ◦ Homeostatic defect in FOB cell survival in spleen ◦ Decreased peritoneal B2 cells • *In vitro* stimulation: ◦ Normal B cell proliferation and survival under anti-IgM stimulation with or without anti-CD40 ◦ Decreased viability after LPS stimulation accompanied by mitochondrial damage • Antibody responses: ◦ TD antigen: OVA + CFA ▪ Normal level of total Ig but reduced Ag-specific IgM in the serum	(66)
	*Atg5^f/f^;CD21-Cre; Fas^lpr/lpr^*	• Lupus disease model: ◦ Decreased number of long-lived PCs ◦ Reduced level of hypergammaglobulinemia and autoreactive antibodies ◦ Fewer IgG depositions in the kidney	(66)
	*Atg5^f/f^;CD19-Cre; Tlr7.1^Tg^*	• Lupus disease model: ◦ Homeostatic defect in FOB cell survival in spleen ◦ Reduced production of inflammatory cytokines ◦ Lower level of autoreactive antibodies ◦ Lower lesion severity scores for glomerulonephritis ◦ Increased viability of mice	(67)
ATG7	*Atg7^f/f^;CD19-Cre*	• B cell development and maturation: ◦ Reduced number of peritoneal B1a cells ◦ Normal B2 cells • Antibody responses: ◦ TD antigen: NP-KLH + Alum ▪ Similar number of GC B cells ▪ Normal level of primary antibodies and similar number of ASCs ▪ Decline in memory B cells 2 weeks after immunization, likely caused by ROS accumulation and lipid peroxidation ▪ Severe reduction in secondary antibody production and ASCs after rechallenge ◦ TD antigen: influenza A virus ▪ Fewer memory B cells 2 months after infection ▪ Reduced level of secondary antibodies and ASCs after rechallenge ▪ Extensive lung destruction and leukocyte infiltration with higher mortality • Pristane-induced lupus model: ◦ Suppressed glomerulonephritis ◦ Reduced prevalence of autoreactive memory B cells	(68, 69)
ATG16L1	*Atg16l1^f/f^;CD19-Cre*	• B cell development and maturation: ◦ Higher proportion of pre-B cells (Hardy fraction D) and lower proportion of mature circulating B cells (Hardy fraction F) ◦ Decreased proportion of MZB cells ◦ Decreased proportion of B1a cells • *In vitro* stimulation: ◦ Comparable levels of B cell division and ASC differentiation to CpG stimulation • Antibody responses: ◦ TD antigen: NP-KLH + Alum ▪ No difference in GC formation and primary antibody production	(16)
WIPI2	*Wipi2* ^p/p^ (aberrant allele)	• B cell development and maturation: ◦ No difference in all B cell compartments except for reduced peritoneal B1a cells	(16)
	Inject a mixture of 80% B cell-deficient μMT and 20% *Wipi2* ^p/p^ BM cells into irradiated μMT mice	• Antibody responses: ◦ TD antigen: NP-KLH + Alum ▪ Significant reduction in GC B cells and diminished percentage of centroblasts ▪ Slight increase of ASCs ▪ Enhanced level of IgM but decreased IgG	(16)
	*Wipi2* ^-/-^	• B cell development and maturation: ◦ Decreased percentage of early B cells in the BM ◦ Normal B2 cell compartment ◦ Decreased peritoneal B1a cells	(16)
	Inject fetal liver cells from *Wipi*2^-/-^ mice into irradiated WT mice	• *In vitro* stimulation: ◦ Increased number of ASCs with CpG stimulation • Antibody responses: ◦ TD antigen: NP-KLH + Alum ▪ Significant reduction in GC B cells and diminished centroblast prevalence ▪ Decreased ASCs ▪ Decreased level of Ag-specific IgM and IgG ▪ Insignificant reduction in memory B cells	(16)
RUBICON	*Rubicon^f/f^;CD19-Cre*	• B cell development and maturation: ◦ Comparable B cell subsets in BM and spleen ◦ Increased number of peritoneal B1a, B1b, and B2 cells • *In vitro* stimulation: ◦ Decreased FOB proliferation with anti-IgM or CpG stimulation ◦ Increased MZB proliferation with CpG, R848 or imiquimod stimulation • Antibody responses: ◦ TD antigen: NP-KLH + Alum ▪ Higher frequency of Ag-specific IgG1 and GC B cells ▪ Decreased number of ASCs and reduced level of high-affinity IgG in the serum ▪ Enhanced memory B cell generation ▪ Higher level of ASCs after rechallenge	(46, 70)
	Inject a 1:1 ratio of *Rubicon* ^-/-^ and WT BM cells into irradiated WT mice	• *In vitro* stimulation: ◦ Slight decrease in proliferation of FOB cells after CpG or anti-IgM stimulation ◦ Increased proliferation of MZB and B1 cells after CpG, R848 or imiquimod stimulation • Antibody responses: ◦ TD antigen: VLPs ▪ Enriched proportion of RUBICON-deficient B cells in GC compartment	(70)
RUBICON and ATG7	*Atg7^f/f^Rubicon^f/f^;CD19-Cre*	• Antibody responses: ◦ TD antigen: NP-KLH + Alum ▪ Normal levels of IgG1 and GC B cells ▪ Decreased number of ASCs ▪ Normal memory B cell generation	(46)

^1^BECLIN1, WIPI2, ATG5, ATG7, and ATG16L1 contribute to both canonical and noncanonical autophagy pathways (30, 33, 34). RUBICON is involved in noncanonical autophagy but also inhibits canonical autophagy (46). When interpreting the phenotypes of autophagy factor mutant mice, it is therefore challenging to distinguish between B cell phenotypes contributed by canonical versus noncanonical autophagy. Please see the main text for further discussion.

^2^Studies from different investigators using the same mouse model are shown separately, as different parameters were studied and/or different stimuli were employed.

^3^B cell Hardy fractions refer to a subclassification of B cell precursors devised by Hardy and colleagues (71).

Ag, antigen; ASC, antibody-secreting cell; BM, bone marrow; CFA, complete Freund’s adjuvant; CGG, chicken gamma globulin; DSS, dextran sodium sulfate; FOB, follicular B; GALT, gut-associated lymphoid tissue; GC, germinal center; Ig, immunoglobulin; KLH, keyhole limpet hemocyanin; lpr, lymphoproliferation; LPS, lipopolysaccharide; MZB, marginal zone B; NP, nitrophenol; OVA, ovalbumin; PC, plasma cell; Rag, recombination activating gene; ROS, reactive oxygen species; TD, T cell-dependent; Tg, transgenic; TI, T cell-independent; TLR, toll-like receptor; TNP, trinitrophenol; VLP, virus-like particle.

### 4.1 B cell development

Autophagy is dynamically regulated to meet the metabolic demands during B cell development, with the maximal levels of metabolic activity occurring in the proliferative early B cell developmental stages, and a decline in quiescent mature B cells (1). Studies using chimeric mice generated by complementing recombination activating gene (*Rag*)-deficient blastocysts with *Beclin1* ^-/-^ embryonic stem cells or by transplanting fetal liver cells from *Atg5* ^-/-^ or *Wipi2* ^-/-^ mice revealed dysregulated pro- to pre-B cell transition (16, 61, 62). However, mice with B cell-specific ablation of *Atg5*, *Atg7*, *Atg16l1*, or *Rubicon* mediated by *Mb1-Cre*, *CD19-Cre*, or *CD21-Cre* demonstrated minimal effects on early B cell development in the bone marrow. The varying outcomes observed in these different models may be caused by the nuanced role of autophagy in hematopoietic cell development, affecting the global but not conditional knockout animals (16, 63, 66, 68). In secondary lymphoid tissues, the absence of ATG5, ATG7, or RUBICON had little impact on the homeostasis of the mature B cell population (46, 64, 68). Again, contrasting findings emerged from other investigators employing the same or different genetic models. A study with *Atg5* ^f/f^;*CD19-Cre* mice observed a decrease in the total B cell population in the spleen, Peyer’s patches, and lamina propria (63). Additionally, similar reductions in spleen FOB cells were reported when *Atg5* was deleted by *Mb1-Cre* that is expressed at an early B-lymphoid progenitor stage and by *CD21-Cre* that is expressed at the mature B cell stage. Notably, *Atg5* ^f/f^ *;Mb1-Cre* mice demonstrated more pronounced outcomes compared with *Atg5* ^f/f^;*CD21-Cre* mice, indicating that the timing of deleting a core autophagy factor differentially influences B cell homeostasis (66). Unlike the discrepancies observed for total B cells and FOB cells, MZB cells remained unaffected in most of the mouse models examined, except for a decrease in their prevalence in *Atg16l1* ^f/f^;*CD19-Cre* mice (16, 64, 68).

Innate-like B1 cells contain a unique metabolic profile characterized by enhanced glucose uptake and oxidative phosphorylation (72–74). Multiple studies have provided evidence that autophagy is required for peripheral self-renewal of B1a cells but not for the development of B1 progenitor cells (66, 75). Autophagy-deficient B1a cells fail to self-renew and contain substantial metabolic disturbances, including dysregulated expression of metabolic genes, decreased fatty acid uptake, and accumulation of intracellular lipid droplets (75). Interestingly, *Rubicon* ^f/f^;*CD19-Cre* mice exhibited elevated counts of peritoneal B1a, B1b, and B2 cells, underscoring the distinct contribution of RUBICON to the development and/or maintenance of B1 cells compared with ATG5, ATG7 or ATG16L1 (46). Given the peritoneum-specific increases in both B1 and B2 cells in *Rubicon* ^f/f^;*CD19-Cre* mice, it is possible that RUBICON inherently facilitates B cell adaptation within the peritoneal cavity.

### 4.2 BCR internalization and MHC class II-restricted antigen presentation

After migrating to secondary lymphoid organs, antigen-specific B cells may recognize antigens either as soluble structures or displayed at the surface of macrophages, dendritic cells or follicular dendritic cells, which can all present antigens to B cells (3). BCR engagement triggers the formation of BCR-dependent immunological synapses to internalize and process the antigen to generate peptide-MHC class II complexes for cognate T cell recognition (76–78). Recent research suggests that ATG5, likely in complex with ATG12 and ATG16L1, is responsible for BCR clustering and internalization of synapse-tethered particulate antigen to MHC class II-containing vesicles (79). This conclusion was supported by studies inhibiting PI3K activity with wortmannin, with the caveat that this reagent is not specific for PIK3C3. However, an ULK1 inhibitor did not impede this process, suggesting the involvement of CASM (79). Consistent with these findings, an additional study found that BCR-mediated antigen uptake utilizes the ATG8/LC3 lipidation machinery (80, 81). Collectively, these studies provide evidence that the ATG8/LC3 conjugation system is activated in response to BCR ligation and targets the internalized antigen for further processing. Antigen recognition also boosts lysosome exocytosis, releasing digestive hydrolases to facilitate antigen extraction from the surface of cells presenting antigens to B cells (82, 83). Upon their recruitment following immune synapse formation, lysosomes fuse with the plasma membrane and secrete their acidic vesicle contents, including the hydrolase cathepsin S, to facilitate antigen extraction and presentation to T cells (84). While the involvement of ATG8/LC3 lipidation in the generation of EVs is established in various mammalian cells, the effects of autophagy-related components on the secretion of B cell EVs are not yet fully understood. Nevertheless, a recent study showed that IL-4- and anti-CD40-stimulated B cells secrete LC3-II-containing EVs in a manner dependent on the small GTPase RAB27a (85). Together, the available studies suggest that autophagy regulators play a significant role in the extraction of B cell-associated antigens, potentially by altering the secretion and function of EVs.

The connection between macroautophagy and MHC class II-restricted antigen presentation was initially demonstrated by the significant proportion of MHC class II ligands sourced from intracellular antigens, including cytosolic and nuclear antigens, in human B lymphoblastoid cells (86–91). Interestingly, nutrient deprivation in these cells resulted in enhanced presentation of intracellular antigens, further indicating the role of macroautophagy in this process (86, 87, 91–93). Moreover, inhibiting PI3K or silencing *Atg7* in human B cells disrupted the intracellular processing of Epstein-Barr virus (EBV) nuclear antigen EBNA1, thereby reducing the display of specific CD4^+^ T cell epitopes from EBNA1 on the B cell surface (94). However, the mechanisms by which such antigens are specifically targeted for presentation by MHC class II on B cells are not well understood. Several autophagy receptors, including P62, NDP52, NBR1, and optineurin, can direct ubiquitinated targets to the autophagosome (95, 96), and these receptors therefore represent possible candidates for targeting intracellular antigens to MHC class II-containing compartments. In addition to these studies providing evidence for macroautophagy-mediated MHC class II presentation of intracellular antigens by B cells, multiple reports have shown that fusing viral or tumor antigens with LC3 boosts their MHC class II presentation by human epithelial or melanoma cells and leads to stronger CD4^+^ T cell responses (93, 97, 98). Yet, these findings have not been explicitly tested in B cells, which will require further investigation.

In addition to macroautophagy, studies with human EBV-transformed B lymphoblastoid cells have provided evidence that CMA contributes to the presentation of cytosolic antigens on MHC class II (88, 99, 100). During CMA, lysosome-associated membrane protein 2A (LAMP-2A), with the assistance of heat shock protein 70 (HSC70), imports cytosolic proteins harboring the pentapeptide KFERQ sequence directly into lysosomal membranes for degradation (24, 101). Overexpression of LAMP-2A or HSC70 resulted in enhanced MHC class II presentation of a peptide derived from the cytoplasmic antigen glutamic acid decarboxylase (GAD) by human B lymphoblastoid cells (99). LAMP-2 isoforms also have a reciprocal modulatory mechanism that can influence the CMA pathway and MHC class II-restricted antigen presentation. Specifically, the LAMP-2C isoform selectively perturbs the MHC class II presentation of cytosolic antigen by blocking CMA-mediated peptide loading onto endosomal MHC class II molecules (102, 103).

### 4.3 B cell activation and differentiation

Autophagy not only influences B lymphocyte development but also controls B cell activation and differentiation. With appropriate stimulation, B cells in lymphoid follicles either differentiate into short-lived ASCs or enter GCs to undergo antibody affinity maturation, which generates either long-lived plasma cells or memory B cells (1–3). Early studies suggested no major involvement of autophagy in short-term B cell activation, as ATG5-deficient B cells displayed comparable proliferation and survival when activated by anti-IgM antibodies with or without anti-CD40 antibodies *in vitro* (66). Moreover, mice with B cell-specific deficiencies in ATG5 or ATG7 exhibited a similar capacity to generate GC B cells as compared to their wild-type counterparts following T cell-dependent (TD) antigen immunization (64, 68, 79). These mutant mice also demonstrated proficiency in undergoing CSR and somatic hypermutation (63, 68, 69). However, subsequent studies revealed a bafilomycin A1 (BafA1)-sensitive autophagy flux in response to BCR stimulation (16). Since BafA1 inhibits the vacuolar type ATPase (V-ATPase) and thus suppresses ATG8/LC3 lipidation during CASM, this finding suggests involvement of CASM in GC B cell activity (44, 104, 105). The engagement of receptors on B cells, most notably interaction of CD40 with T cell-expressed CD40L, triggers the activation of transcription factor NF-κB, which directly induces the expression of activation-induced cytidine deaminase (AID) that is crucial for CSR in the immunoglobulin heavy chain locus (106, 107). Co-localized with CD40, RAB7, a small GTPase that regulates endosome maturation and autolysosome formation, directly interacts with signal adaptor tumor necrosis factor receptor-associated factor 6 (TRAF6) E3 ubiquitin ligase to upregulate the activity of TRAF6 and enhance NF-κB activation (108). In particular, RAB7 also interacts with RUBICON to regulate both the endocytic and autophagic pathways, suggesting a role for CASM in B cell signaling (109). The possible involvement of noncanonical autophagy was further investigated using mice with B cell-specific RUBICON-deficiency. Immunization of mice transplanted with a 1:1 mixture of RUBICON-deficient and wild-type bone marrow cells with virus-like particles (VLPs) showed significant enrichment of mutant B cells in the VLP^+^ GC compartment, suggesting that RUBICON-deficiency provides a competitive advantage to GC B cells (70, 110). Additionally, *Rubicon* ^f/f^;*CD19-Cre* mice displayed increased levels of antigen-specific IgG^+^ GCs following TD antigen immunization, suggesting a unique role for noncanonical autophagy in antibody affinity maturation and isotope switching. However, *Atg7^f/f^Rubicon^f/f^*;*CD19-Cre* double knockout animals displayed normal GC formation, indicating that enhanced autophagy was responsible for the increase in antigen-specific IgG^+^ GCs in *Rubicon* ^f/f^;*CD19-Cre* mice (46).

More distinct outcomes were observed in the primary antibody response to TD antigen immunization and in the context of primary infection. *Atg5^f/f^*;*CD19-Cre* mice exhibited reduced levels of both antigen-specific IgM and IgG antibodies following trinitrophenol (TNP)-chicken gamma globulin (CGG) and nitrophenol (NP)-CGG immunizations (63, 64), whereas *Atg7^f/f^*;*CD19-Cre* and *Atg16l1^f/f^*;*CD19-Cre* mice did not display such decreases after NP-keyhole limpet hemocyanin (KLH) immunization (16, 68). This variability may possibly have arisen due to the utilization of diverse antigens and/or adjuvants, or alternatively, due to distinct functions of the autophagy factors investigated. Moreover, mice with B cell-specific ATG7-deficiency exhibited diminished levels of antigen-specific antibodies as time progressed, suggesting the potential significance of ATG7 in maintaining ASC populations (68). These findings highlight the nuanced and factor-specific impact of autophagy-related gene ablation on antibody production in response to distinct antigens. Particularly, compromised BCR clustering was noted exclusively upon stimulation with particulate antigens rather than soluble ones, illustrating that various antigens can elicit distinct responses (79, 111). In addition, the switch from canonical autophagy to noncanonical autophagy during the GC reaction may also explain the paradox that autophagy contributes to BCR trafficking and MHC class II-restricted antigen presentation but shows limited effects on TD antibody responses, suggesting that alternating autophagy routes can influence B cell responses to antigen exposure (16). Moreover, prior to their secretion, antibodies are routed from the ER to the Golgi apparatus where they are glycosylated (112, 113). Recent evidence indicates that one member of the SNARE family, SEC22B, is involved in this constitutive exocytosis pathway. *Sec22b^f/f^*;*Mb1-Cre* mice displayed reduced circulating antibodies at both steady state levels and after TI antigen immunization, exacerbated unfolded protein response (UPR), and morphologically disturbed ER and mitochondria, all of which suggests SEC22B as an essential regulator of plasma cell fitness and humoral immune responses (114).

In additional to BCR-mediated stimulation, B cells can be activated by engagement of toll-like receptors (TLRs) by pathogen-associated molecular patterns, which induce B cell proliferation and differentiation into ASCs (115). Stimulation of ATG5-deficient B cells isolated from *Atg5* ^f/f^ *;CD19-Cre* mice with a TLR4 agonist (lipopolysaccharide [LPS]) or a TLR9 agonist (CpG oligodeoxynucleotides) led to impaired differentiation into ASCs *ex vivo*. Consistent with these findings, these cells downregulated the expression of multiple genes, including *Prdm1* (encoding the transcriptional repressor BLIMP1), *Sdc-1* (encoding the regulator of cell behavior SYNDECAN-1/CD138), and *Xbp-1* (encoding the stress-induced transcription factor XBP-1), that play critical roles in plasma cell differentiation (63). However, these findings were not recapitulated by all studies. Upon depletion of ATG5 in mice with different *Cre* drivers (*Mb1-Cre* and *CD21-Cre*) B cells showed decreased survival with normal expression of SYNDECAN-1/CD138 and increased mitochondrial content, suggesting autophagy participates in early plasma cell survival by limiting mitochondrial stress (66). Mediated by AMPK, ULK1 migrates to the mitochondria and triggers a signaling cascade that leads to the recruitment of the ATG8/LC3 machinery to support mitochondrial clearance and quality control in B cells (23, 66, 116–118). After LPS stimulation, plasma cells from *Atg5* ^f/f^ *;CD19-Cre* mice displayed ER enlargement, upregulation of *Prdm1* and *Xbp-1* gene expression, and increased IgM production, demonstrating that proficient autophagy confers a selective advantage on differentiating plasma cells (64). Another study further showed that this reduced proliferation of ATG5-deficient FOB cells occurs only in response to TLR7 or TLR9 stimulation but not TLR4 stimulation, whereas MZB cells with the same deficiency showed increased proliferation in response to activation by all three TLRs, with induction of mitogen-activated protein kinase (MAPK), IκB kinase (IKK), and NF-κB (65). Furthermore, MZB cells from RUBICON-deficient mice showed impaired LC3 lipidation and enhanced proliferation upon stimulation with TLR7 or TLR9 ligands, relative to control cells (70). This suggests that RUBICON plays a role similar to ATG5 in regulating MZB cell responses to specific TLR ligands (65, 70). This discrepancy between FOB and MZB cells may explain the discordance between different studies and underline the importance of distinguishing B cell subsets when exploring B cell autophagy (70). *In vivo* experiments with ATG5-deficient mice showed a reduction in serum antigen-specific IgM antibodies in response to immunization with the TI antigens TNP-LPS, TNP-Ficoll, or the pneumococcal vaccine pneumovax (63, 64). A notable exception was that immunization with NP-Ficoll resulted in higher titers of anti-NP immunoglobulin and ASCs two weeks after immunization, for reasons that remain unclear (64). While there has been limited exploration into the role of CASM during TLR stimulation in B cells, LANDO has been found to enhance TLR4 recycling in primary microglia, where it facilitates amyloid-beta (Aβ)-associated phagocytosis (52, 119). Whether similar processes are at play in B cells remains to be explored.

### 4.4 B cell memory and long-term humoral immunity

Two major components of long-term humoral immunity include long-lived plasma cells and memory B cells. Long lived plasma cells reside in the bone marrow and secrete antibodies to neutralize pathogens immediately upon reinfection (3). *Atg5* ablation via *CD19-Cre* compromised long-term maintenance of plasma cells (64, 66). The available evidence indicates that ATG7 is dispensable for memory B cell generation (68). Transcriptionally, autophagy genes do not increase until 4 to 8 weeks after TD immunization, and high levels of ATG8/LC3 aggregation in memory B cells are not identified until 8 weeks after immunization (68). Nevertheless, ATG7-deficient memory B cells display reduced capacity to form ASCs upon antigen rechallenge. This was associated with increased death of mutant memory cells, which was prevented by inhibiting oxidative stress with NecroX-2 or α-Tocopherol, or by scavenging ROS with N-acetyl-L-cysteine (NAC) or Tempol, indicating the importance of autophagy in metabolic homeostasis of memory B cells (68). Members of the forkhead box (FOXO) family of transcription factors, especially FOXO1 and FOXO3, regulate autophagy at the epigenetic level, which is upregulated in memory B cells. Silencing FOXO3 in mice decreased the B cell expression of *Beclin1* and *Ulk1*, and impaired memory responses upon antigen rechallenge (69). However, given the potential variations in results when depleting different autophagy factors, it is crucial to interpret findings obtained from B cell-specific ATG7-deficient mice with caution. Further investigation into the functions of other autophagy regulators in memory B cells is warranted.

A recent study suggested the potential role of CASM in memory B cell functions. *Rubicon^f/f^*;*CD19-Cre* mice contain significant increases in antigen-specific memory B cells and ASCs after antigen rechallenge, indicating that RUBICON suppresses the formation of memory B cells and ASCs (46). Similar to GC B cells, the increase in memory B cells was attenuated in *Atg7^f/f^Rubicon^f/f^*;*CD19-Cre* double knockout mice, indicating that increased macroautophagy induced by RUBICON-deficiency is responsible for promoting memory B cell differentiation (46).

### 4.5 B cell cytokine secretion

In addition to their capacity to produce antibodies and present antigens to MHC class II-restricted T cells, B cells can produce immunomodulatory cytokines, including pro-inflammatory cytokines such as IL-6 and tumor necrosis factor (TNF)-α and immunosuppressive cytokines such as IL-10 that can promote or protect against disease (120).

While not yet investigated in B cells, autophagy suppresses inflammatory cytokine production by phagocytes via multiple mechanisms: a) preventing inflammasome activation by removing mitochondria (121, 122), b) targeting inflammasome components and cytokines for degradation (123, 124), c) preventing constitutive immune cell signaling by stalling cargo degradation in LAP or reducing receptor recycling in LANDO (54, 125), d) inhibiting the assembly of essential cytokine signal transducers, stimulators of interferon genes (STING), and TANK-binding kinase 1 (TBK1), that are required for cytosolic DNA-triggered innate immune responses (126), and e) enhancing the degradation of cytoplasmic DNA to prevent excessive cGAS-STING activation (127).

The regulatory capacity of B cells is highlighted in the context of IL-10-producing regulatory B cells (Bregs), known for their critical function in modulating immune responses (128). Studies have indicated that dual stimulation via TLR9 and CD40 for 48 hours is optimal for inducing and differentiating IL-10-producing Bregs (129). As autophagy enhances B cell differentiation and proliferation in response to TLR9 stimulation, autophagy might be expected to play a critical role in Breg cell differentiation (65). This notion is further supported by observations that, following BCR engagement, TLR9-containing endosomes are recruited to autophagosomes, which contain the internalized BCR-antigen complex. This co-localization is disrupted by the PI3K inhibitor 3-methyladenine (3-MA), suggesting potential interactions between BCR engagement, TLR9 signaling, and autophagic pathways in Bregs (80).

## 5 Impact of B cell autophagy on health and disease

Due to the pleiotropic role of autophagy in immune and non-immune cells, mutations in autophagy factors have been linked to multiple human diseases, including cancer, chronic inflammation, cardiovascular diseases, infection, neurodegeneration, immune system disorders, and aging (130).

The clinical significance of autophagy in B cells primarily manifests in its involvement in adaptive immunity, including protective immunity against infection and the development of autoimmunity (13, 14, 88, 92, 131–133). In models of influenza virus infection, deletion of *Atg7* in B cells via *CD19-Cre* led to the loss of memory B cells and impaired secondary responses (66). Additionally, B cells from *Atg5* ^f/f^;*CD19-Cre* mice exhibited reduced antibody production and impaired protection against the helminth parasite *Heligmosomoides polygyrus* and reduced severity of dextran sodium sulfate-induced colitis (63).

Compromised adaptive immunity is a major hallmark of aging (134). One recent study linked autophagy deficiency to impaired memory B cell responses and poor vaccination efficacy in elderly individuals (134). More importantly, treatment with the autophagy inducer spermidine was able to reverse memory B cell senescence in old mice (134), underscoring the therapeutic potential of autophagy intervention for age-related diseases.

Deletion of *Atg5* via *CD21-Cre* or *CD19-Cre* causes a notable reduction in autoantibody production and kidney inflammation in genetic mouse models of systemic lupus erythematosus (SLE) (66, 67). Similarly, these hallmarks of lupus failed to manifest in *Atg7^f/f^;CD19-Cre* mice treated with the hydrocarbon oil pristane (67, 131). Consistent with these findings, dysregulation of macroautophagy and CMA have been described in B cells of lupus patients (133, 135). Autophagy has also been implicated in the pathogenesis of rheumatoid arthritis (RA). Classic pro-autophagic stimuli, such as starvation, facilitated B cell-associated MHC class II-mediated presentation of citrullinated peptides, which are prominently involved in RA (136). This enhanced presentation of citrullinated peptides was diminished by the autophagy inhibitor 3-MA or reduced *Atg5* expression (136). Since the presentation of peptides derived from self-antigens is crucial for the development of self-tolerance, these findings suggested a protective role of autophagy in RA development (136, 137).

Autophagy’s role in adaptive immune responses offers therapeutic potential, particularly in enhancing vaccine efficacy in the elderly, and may hold promise for treating autoimmune disorders.

## 6 Conclusions and future directions

The studies reviewed here provide strong evidence that macroautophagy plays a critical role in B cell biology, intersecting with B cell development, BCR signaling, MHC class II antigen presentation, GC formation, antibody production, plasma and memory cell differentiation, and disease (summarized in **Figure 2** and **Table 1**). It is now well appreciated that components of the macroautophagy machinery are utilized for other intracellular vesicle trafficking processes that are instrumental in the development and functionality of B cells (84). While offering explanations for inconsistencies found in previous studies, the newly identified role of CASM in B cells has generated further questions (138). ATG16L1-deficient mouse models could serve as valuable tools for differentiating between macroautophagy and CASM. In the process of macroautophagy, ATG16L1 associates with FIP200 and WIPI2 through its central FIP200-binding domain. Conversely, the WD40 domain of ATG16L1 is crucial for noncanonical autophagy but dispensable for macroautophagy (139). Future research may focus on comparing outcomes from these two models, particularly in the context of GC formation and primary immune responses, to unravel the distinct contributions of macroautophagy and CASM to B cell function. Furthermore, additional studies are needed to explore the role of canonical and noncanonical autophagy to the presentation of MHC class II-restricted antigens by B cells, particularly in relation to ATG8/LC3 lipidation. To date, only a small selection of antigens has been studied, and those investigations have predominantly utilized cell lines transformed with EBV, which modulates autophagy (94). Exploring antigen targeting through various autophagy regulators or ATG8/LC3 lipidation might be a fruitful avenue for future exploration, as initially indicated by reports employing ATG8/LC3 fusion antigens. Specifically, focusing on the intracellular expression of these targeted antigens in B cells might enhance the precision of T cell activation and antibody production against infectious agents and tumors (97, 98, 140). Lastly, autophagy has been implicated in the survival and functionality of self-reactive B cells in autoimmune diseases (13, 66, 141). Future research may concentrate on how B cell autophagy contributes to the MHC class II-mediated presentation of autoantigens to CD4^+^ T cells, the benefits of autophagy to B cell apoptotic or metabolic stress, and its impact on T cell evasion of tolerogenic controls. Collectively, these studies will be helpful for designing autophagy-based therapies of human disease that target B cells. Pharmacological agents that selectively target autophagy are under development (141).

## Author contributions

YW: Writing – original draft, Visualization, Writing – review & editing, Conceptualization. LW: Writing – review & editing. LVK: Supervision, Conceptualization, Writing – review & editing.
